# *Actinomyces meyeri*-induced brain abscess in pregnancy: a case report

**DOI:** 10.1186/s12883-023-03453-7

**Published:** 2023-11-11

**Authors:** Yaping Zhang, Zhinan Ye, Qianqian Miao, Hao Xu, Wenyang Pang

**Affiliations:** 1 Department of neurology, Taizhou Municipal Hospital, Taizhou, 318000 Zhejiang Province China; 2 Department of Oncology Surgery, Taizhou Municipal Hospital, Taizhou, 318000 Zhejiang Province China

**Keywords:** *Actinomyces meyeri*, Brain abscess, Pregnancy, Case report

## Abstract

**Background:**

Brain abscesses can occur when suppurative, bacterial or protozoan infections spread to the brain. Here, we report a rare case of *Actinomyces meyeri*-induced brain abscess in a pregnant woman.

**Case presentation:**

We present the case of a 38-years-old primipara admitted to the emergency department at our hospital with a 4-day history of fever and vomiting. The symptoms worsened rapidly during the 8 h prior to admission, and the patient experienced a sudden loss of consciousness 4 h before arrival to the unit. Brain magnetic resonance imaging revealed abnormal signals in the right parietal–temporal lobe, suggesting the possibility of abscess rupture into the ventricle and sulcus. Right lateral ventricle compression and midline structure deviation to the left were noted. A right temporal–occipital mass with midline shift was detected. Emergency procedures were promptly performed, including craniotomy, removal of the right temporal–occipital mass, decompressive craniectomy, implantation of an intracranial pressure monitoring device, and external ventricular drainage. Cerebrospinal fluid culture indicated infection with *Actinomyces meyeri.* After administration of antibiotics, including linezolid and meropenem injections, along with treatments to decrease intracranial pressure, the patient’s vital signs stabilized. However, the patient developed hydrocephalus, requiring placement of a hydrocephalus shunt several months later. Throughout this period, the patient remained in a coma vigil state, and labor was induced for the fetus.

**Conclusions:**

Although the patient did not present with any apparent predisposing causes for brain abscess, a scout view of CT revealed dental caries. In addition, the occurrence of the brain abscess may have been influenced by the hormonal changes during pregnancy, including increased secretion of estrogen and progesterone, as well as decreased immune function. Early diagnosis and intervention are crucial in such cases. Therefore, it is recommended to seek early medical attention if symptoms such as fever, vomiting, and changes in mental state occur during pregnancy, as the prognosis for both the mother and infant is poor once the abscess ruptures.

**Supplementary Information:**

The online version contains supplementary material available at 10.1186/s12883-023-03453-7.

## Background

Brain abscess is a condition characterized by the formation of an abscess within the cerebral parenchyma due to suppurative infections reaching the brain. Although less common, brain abscesses can also result from fungal or protozoan infections. These abscesses commonly occur secondary to rhinogenic, odontogenic, and otogenic infections, as well as post-trauma and post-endovascular interventional procedures. Brain abscesses can occur at any age but are more prevalent among young adults. The incidence of brain abscesses during pregnancy is low, and *Actinomyces meyeri*-induced brain abscess rupturing into the ventricle during pregnancy is even less common, with no previous reports of such a case available in the literature to date. The symptoms of brain abscesses are usually nonspecific, and the condition is severe, posing a significant threat to the lives of both the mother and the infant. Consequently, the mortality rate is exceedingly high. Here we report a case of a pregnancy-associated brain abscess caused by *Actinomyces meyeri*.

## Case presentation

The patient was a previously healthy 38-year-old primipara at 8 weeks of amenorrhea. She was admitted to the emergency department at our hospital due to a 4-day history of fever and vomiting that exacerbated in the 8 h prior to admission. The patient experienced a sudden loss of consciousness 4 h before arrival to the unit. On physical examination, the patient had a body temperature of 39.0 °C, blood pressure of 131/69 mmHg, and a heart rate of 80 beats/min. The patient was in a coma with a Glasgow Coma Scale score of 6 (1 + 1 + 4). Pupillary examination revealed a diameter of 0.4 cm and 0.2 cm for the right and left pupils, respectively, with neither showing direct nor indirect light reflexes. Cardiac examination did not reveal any significant abnormalities. Her abdomen was flat and soft, without tenderness or noticeable abdominal muscle tension. No movement was observed in the limbs, and there was no voluntary motor response. Her leukocyte count was slightly elevated at 16.6 × 10^9^/L, while the C-reactive protein concentration was within the normal range.

**Fig. 1:**
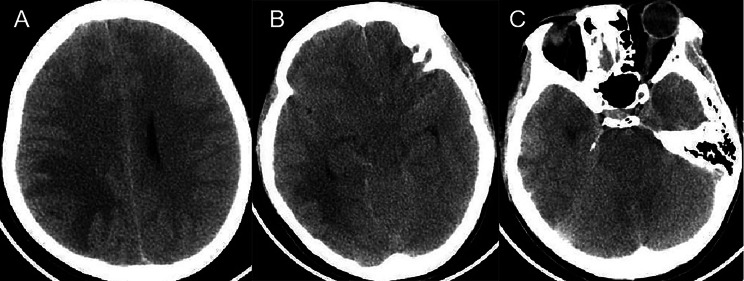
Axial cut of CT scan of the brain. **A**: A patchy low-density shadow was observed in the right temporal–parietal lobe. The right lateral ventricle was compressed and flattened. **B**:The suprasellar cistern was compressed and narrowed, displaying an irregular shape. Brain herniation needed to be excluded. **C**: The brainstem was compressed, and the boundaries were unclear. Brain swelling may have occurred

**Fig. 2:**
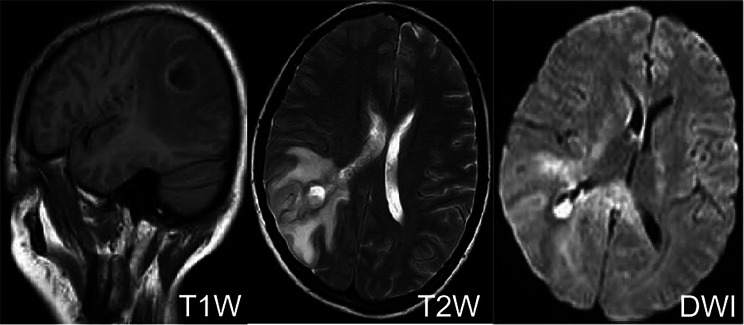
Magnetic resonance images of the brain abscess. TIW = T1-weighted; T2W = T2-weighted; DWI = diffusion weighed imaging. A patchy abnormal signal was observed in the right parietal–temporal lobe, with a low signal on T1-weighted imaging surrounded by annular high signals, and a high signal on T2-weighted imaging surrounded by annular low signals around the lesion. Diffusion-weighted imaging showed high signals with patchy long T1 and long T2 signals around the lesion; the lesion appeared to be connected to the right lateral ventricle

Cranial computed tomography (CT) revealed a large low-density shadow in the right parietal–temporal lobe, suggestive of cerebral swelling. The suprasellar cistern appeared compressed and narrowed, displaying an irregular shape, although brain herniation still needed to be ruled out (Fig. 1). Cranial magnetic resonance imaging (MRI) revealed abnormal signals in the right parietal–temporal lobe, which may have been due to an abscess rupturing into the ventricle and sulcus. The presence of a tumor had yet to be excluded. There were signs of increased intracranial pressure, with compression of the right lateral ventricle and a shift in midline structures to the left. Additionally, a mass was identified in the right temporal–occipital region, accompanied by a midline shift and severe cerebral edema (Fig. 2).

Emergency interventions were initiated, including the administration of mannitol (150 ml) and dexamethasone (5 mg) injections to treat cerebral edema and reduce intracranial pressure, respectively. Empirical antibacterial treatment with ceftriaxone sodium injections (2 g) was initiated. A lumbar puncture was successfully performed, revealing cerebrospinal fluid (CSF) pressure above 300 mmH_2_O. The CSF sample was sent for bacterial examination, and the results indicated a bacterial infection. The CSF sample had a reddish, turbid appearance. Routine biochemistry in the CSF revealed that glucose levels were 4.29 mmol/L (normal range: 2.2–3.9 mmol/L), and protein levels were 554 mg/dL (normal range: 15–45 mg/dL). The leukocyte count was 150/µL (normal range: 0–10/µL), with 70% neutrophils and 30% lymphocytes observed.

Upon completion of preoperative preparations, emergency procedures were performed, including examination with craniotomy and removal of the right temporal–occipital mass, decompressive craniectomy, implantation of an intracranial pressure monitoring device, and external ventricular drainage. During the operation, approximately 7 mL of yellowish pus was aspirated. Post-operative pathology revealed a brain abscess, purulent inflammation of the brain tissue. The CSF culture identified the presence of *Actinomyces meyeri*. However, no drug sensitivity test was performed.

After surgery, the patient was administered a regimen of linezolid injections at a dose of 0.6 g q12h, meropenem injections at 2 g q8h, and mannitol injections at 150 mL q8h to mitigate intracranial pressure. The patient’s vital signs remained stable post-operation. After two weeks, the treatment was supplemented with intravenous drip of ornidazole at 0.5 g q12h for anti-infection purposes. Two months post-operation, the treatment was continued with linezolid tablets at a dosage of 0.6 g q12h orally for four weeks for anti-infection purposes. During hospitalization, the outcome of multidisciplinary consultations was that pregnancy should be terminated at an appropriate time following further control of the systemic infection. With informed consent from the patient’s family, a B-ultrasound-guided intra-amniotic injection of rivanol was performed, resulting in the intact delivery of the fetus.

**Fig. 3:**
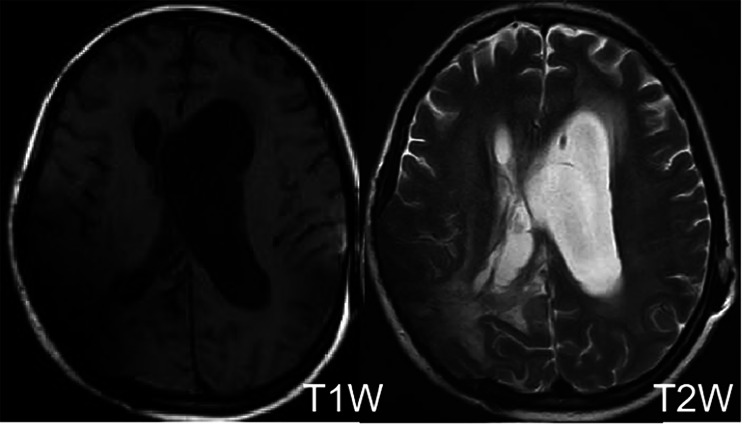
Magnetic resonance images of the brain abscess. TIW = T1-weighted; T2W = T2-weighted. Patchy abnormal signals were observed in the right occipital lobe, with low signals on T1-weighted imaging and high signals on T2-weighted imaging, possibly indicating post-operative changes. Formation of local encephalomalacia and post-operative changes of communicating hydrocephalus were observed

**Fig. 4:**
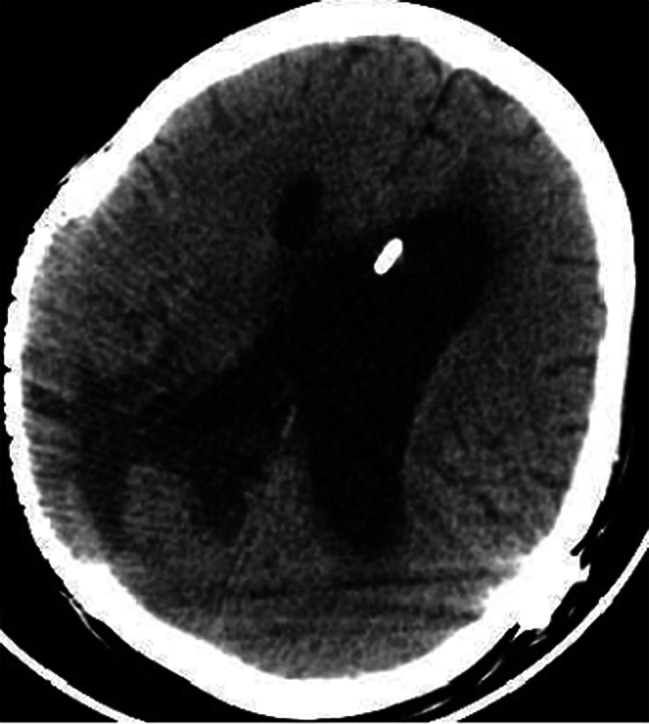
Post-operative changes were observed in the right skull and left frontal bone. Patchy low-density shadows were observed in the right parietal–occipital lobe with unclear boundaries (axial cut of CT scan of the brain)

Following the surgery, the patient exhibited obvious signs of hydrocephalus, and a hydrocephalus shunt was performed several months later. Six months post-craniotomy, a follow-up cranial MRI (Fig. 3) was conducted. A follow-up cranial CT scan (Fig. 4) was also performed one year after the surgery. Both examinations indicated no recurrence of the brain abscess. However, the patient remained in a coma vigil state.

## Discussion

Brain abscess is a rare complication during pregnancy, with only 20 cases reported in the literature in English to date [1–20]. The clinical symptoms, abscess location, etiology, antibiotic treatment, delivery method, maternal and infant morbidity, and mortality rates of these cases are shown in Table 1. Clinical manifestations of brain abscesses during pregnancy are often non-specific and related to the size, number, and location of the lesions; to pathogen toxicity, and to the immune response of the host. Mental state changes are commonly observed, while only 20% of patients with brain abscess display typical symptoms such as fever, headache, and focal neurological deficits. The abscesses are commonly located in the frontotemporal lobe, followed by the parietal lobe, occipital lobe, and brainstem. Manifestations of the abscess are often associated with increased intracranial pressure caused by the mass, which can lead to neurological symptoms. The most concerning complications include brain herniation and abscess rupture. In this particular case, the patient initially exhibited only minor symptoms, including low fever and vomiting. Unfortunately, due to the fact that the patient was experiencing an early pregnancy and that the initial symptoms of brain abscesses exhibit some similarities with common early pregnancy reactions, they were overlooked. As a result, the patient did not receive timely medical attention, leading to a missed opportunity for optimal treatment. Eventually, rapid progression of the condition led to the rupture of the abscess into the ventricles and brain herniation. To the best of our knowledge, this is the first reported case of *Actinomyces meyeri*-induced brain abscess during pregnancy.

**Table 1:** Reported cases of pregnancy-associated brain abscess in the medical literature

author/year	Age(year)/gestational age	preliminary sympt om and sign	Location of brain abscess	source of infecti on	culture	Types of antibiotics	Surgical treatment	Maternal and infant outcome
Raskind R/1966	24/28 W	HA, speech dysfunction, left foot weakness	Frontal lobe	-	Anaerobic β Streptococcus	Tetracycline,procaine penicillin	Abscess aspiration by craniotomy	minimal residual right cranial nerve weakness at 11 months follow-up/good(Vaginal delivery)
Brahan J/1967	ND	HA, confusion	R temporal	R mastoiditis	ND	Surgical drainage	ND	ND
Kahn HS/1975	31/ND	Dysphagia, R-sided weakness	L cerebral hemisphere	IUD (safe T-coil), incomplete abortion	Enterobacter cloacae	None	None	Death/First-trimester spontaneous abortion
Martinez AJ/1980	26/ND	Fever, HA,seizures, L hemiplegia, coma	R frontotemporal,occipital lobes	Corticosteroids	Acanthamoeba culbertson	ND	ND	Death/ND
Wolf P/1983	17/37 W	HA, nausea, vomiting	R frontal lobe	-	Pseudomonas species (contaminant)	ampicillin IV	ND	Death/Normal infant (term cesarean)
Opsahl MS/1983	33/29 W	HA, fever, myalgia, confusion, nuchal rigidity	L parieto-occipital lobe	Sarcoidosis	Nocardia species	ND	surgical drainage by craniotomy	Alive and well/Preterm Birth(Low forceps delivery)
Braun TI/ 1991	25/16w	L-sided HA, mildly confusion, R-sided neurologic signs	L occipital lobe	None	Nocardia asteroids	Sulfisozazole, ampicillin, ceftriaxone	Left occipital craniotomy	Good recovery/Preterm birth,good(Caesarian section)
Cihangiroglu M/2001	25/25 W	R sixth cranial nerve palsy	R cerebral hemisphere (multiple)	Infectious cerebral vasculitis	Aerobic and anaerobic organisms, including Peptostreptococcus	ND	Craniotomy and decompression	ND/ND
Baxi LV/ 2001	36/10w	R hemiparesis, aphasia, DIC	L basal ganglion	Thromphilia	Propienobacterium acnes, staphylococcus capitis	Cefotaxime, ceftazine, vancomycin	None	Residual hemiparesis amenorrhea/good(Low forceps delivery)
Wax JR/ 2004	ND/ 36 W	HA confusion seizure	L temporal lobe	Sinusitis	negative	Cefepime, vancomycin, metronidazole	None	Good recovery/good(cesarean delivery)
Liu SSH/ 2004	32/35w	stuffy nose, rhinorrhea, HA, bilater al eyebal l soreness and low-grade fever	L frontal lobe	Sinusitis	β-hemolytic streptococcus	metronidazole, penicillin G sodium, gentamycin, metronidazole	Drainage	Hydrocephalus hemiparesis, diabetes inspidus/Good(Caesarian section)
Kim HC/ 2007	38/30 W	HA, sinus infecti on, meningeal sign, sudden deterioration of (VA) AT 34 W	Pituitary	Sinusitis	Streptococcus viridians	ceftriaxone,amikacin	Transphenoidal microsurgic al removal	Good recovery/LBW(Emergency Caesarian section)
Jacob CE/ 2009	23/35w	hronic otitis media, fever, HA, gait ataxia	L cerebellar hemisphere	Otitis	Pseudomonas aeruginosa	Penicillin, ciprofloxacin, TMP/SMX	Partial excision of the abscess, modified radical mastoidectomy	Dry left ear, with no residual hearing/good(Caesarian section)
Salvi N /2010	19/39w	Severe HA, facial swelling,mental status	frontal subdural empyema	ND	ND	ND	A right frontal craniotomy with evacuation of empyema	ND/ND(Emergency Caesarian section)
Hobson DT /2011	35/21w	HA, facial swelli ng, mental status changes	L fronta l, tempo Ral, parietal lobe	Infected tooth	Bacteroides fragilis, Wolinella species, Campylobac ter gracilis, Prevotella buccae	Ampicillin, cefotaxime, metronidazole	Drainage, lobectomy	Broca’s aphasia and apraxia with right hemiplegia/good(ce sarean delivery)
Yoshida M/ 2013	24/22w	-	R frontal lobe	none	MSSA	Cefotaxime, meropenem, amoxicillin	None	Good recover/Good(Vaginal delivery)
Bulthuis VJ/2015	36/-	HA, nausea, vomiting	brain stem absces	recurring skin abscesses	Streptococcus constellatus	ceftriaxone, metronidazol e, and fluoxetine) for 12 weeks.	acute stereotactic aspiration of a brainstem abscess	favorable outcome/spontaneou s abortion
Jendoubi A/2016	23/32w	HA, blurry vision and epigastric pain, generalized tonic-clonic seizures	R parieto- occipital lobe juncti on	None	negative	cefotaxime metronidazole,fosfomycin	stereotactic aspiration of the abscess	Epilepsy, homonymous hemianopia/Preterm birth,good(Emergency Caesarian section)
Bahrami R/20 17	25/28w	HA, nausea and vomiting, sudden loss of consciousne ss.	R frontal	sinusitis	negative, but smear was gram positive cocci	ND	emergency large decompres sive craniotomy	died after surgery/died because of preterm delivery(Caesarian section)
our case	35/8w	Fever with vomiti ng, sudden loss of consciousness	R tempo Ral,parietal lobe	none	Actinomyces meyeri	Vancomycin, meropenem, ornidazole	Craniotomy, Drainag e	Vegetative state/Induce labor

W:week; ND: Not described; HA = headache; L = Left; R = Right; DIC: disseminated intravascular coagulation; IUD: Intrauterine device; LBW: low birthweight; TMP/SMX: trimethoprim/sufamethaxazole; MSSA: Methicillin sensitive staphylococcus aureus

Brain abscesses associated with pregnancy predominantly originate from pyogenic infections at neighboring sites. These include rhinogenic, otogenic, and odontogenic brain abscesses, as well as hematogenous, traumatic, and cryptogenic brain abscesses. Rhinogenic brain abscesses account for approximately two-thirds of all brain abscesses, and are primarily caused by *Streptococcus viridans* and β-streptococcus. In contrast, otogenic brain abscesses are often attributed to proteobacteria and anaerobic bacteria. Hematogenous brain abscesses are most frequently caused by *Staphylococcus aureus*, while traumatic brain abscesses are commonly caused by *Staphylococcus aureus* and Enterobacteriaceae bacteria. Our patient, who was previously healthy and 8 weeks pregnant, had no medical history of congenital heart disease, chronic otitis media, or arteriovenous fistula of the lungs. Moreover, the patient had not used steroids, and all her regular prenatal check-ups had shown no abnormalities. However, the emergency scout view of CT revealed dental caries in the second molar. Thus, the possibility that the infection may have originated from this source cannot be ruled out. Previous literature indicates that approximately 7% of brain abscesses are caused by dental infections and procedures. Dental conditions can lead to systemic diseases and other severe complications, especially during pregnancy, when immune function is compromised [17]. In this specific case, the pus culture suggested an uncommon *Actinomyces meyeri* infection.

*Actinomyces* is a genus of anaerobic gram-positive filamentous bacteria commonly found in the oral cavity and the upper respiratory, gastrointestinal, and genitourinary tracts. Endogenous infection may occur in individuals with a compromised immune system or poor oral hygiene, or after tooth extraction or oral mucosal damage, leading to actinomycosis. This condition typically affects the face and neck, and can also extend to the gastrointestinal tract and lungs, leading to infection of these structures [21]. Typical characteristics include a hidden course of the disease, formation of thick wood-like abscesses, presence of sulfur-like granules, and fistula formation [21]. *Actinomyces meyeri* is one of many *Actinomyces* species found in the human microbiota. It was first isolated in 1911 from a patient with pyothorax, and has been reported in the literature in English only 30 times to date [22].

*Actinomyces meyeri* exhibits a tendency to invade and disseminate diseases [1]. These infections are strongly associated with the presence of dental plaque and caries [1]. The bacterium can penetrate deep tissues and readily invade the human central nervous system, leading to severe infections [21, 23]. However, our understanding of the specific virulence mechanisms used by *Actinomyces meyeri* to achieve tissue invasion remains limited. To date, only ten cases of brain abscesses associated with *Actinomyces meyeri* infection have been reported in the literature [22–32], and these are summarized in Table 2.

**Table 2:** Published cases of A. meyeri cerebral abscesses

author/year	Age(year)/sex	preliminary symptom and sign	Location of brain abscess	source of infection	Other cultured organisms	antibiotic	Surgical treatment	outcome
Dijkmans BA/1984	28/F	HA, fever, AMS, meningismus, R-sided hemiparesis	L parietal lobe brain abscess, ventriculitis	Unknown	Streptobacillus moniliformis	Benzathine penicillin 24 mill U x 1 m then 36 mill U + dexamethasone x 1 m	Burr hole drainage, recurrent percutaneous punctures for external drainage	No recurrence at 1 year
Kuijper EJ/1992	44/M	1 month R-sided weakness and dysarthria	L fronto-parietal lobe abscesses (x2), R occi pital lobe absc ess	Unknown	Actinobacillus actinomycentemcomitans	Amoxicillin 6 w, amoxicillin x 12 m	Stereotactic brain biopsy and drainage	Clinical cure; follow-up period not specified
Park HJ/2014	46/M	3d HA and aphasia	L lung mass, L fronto-parietal lobe brain abscess	Unknown	propionibacterium acnes, Fusobacterium nucleatum	penicillin 4 mill Ux 4 w, metronidazole x 4 w, amoxicillin x 11 m	Stereotactic brain biopsy	Resolution of symptoms and significant reduction in mass size at 5 m
Fernandez-Valle T/2014	57/M	Hours AMS and new onset seizure	L parietal lobe abscess	Dental procedure week prior	not reported	Ceftriaxone and metronidazole x unknown duration, amoxicillin x 12 m	Stereotactic brain biopsy	No recurrence at 1 year
Clancy U/2015	55/F	2d HA, R hemisensory loss, unsteady gait	L parietal lobe brain abscess	Dental extraction 7d prior	Group B streptococcus, Staphilococcus capitis	Vancomycin x 11 d, metronidazole x 1 m, Ceftriaxone x 4 m, amoxicillin x 6 m	Craniotomy and drainage	No recurrence at 4 m
Rolfe R/2016	50/M	Unknown	Brain abscess	Pneumonitis	Actinobacillus, mixed anaerobic flora	Ceftriaxone x 1 m, penicillin x5 m	Brain biopsy	Lost to follow-up
Rolfe R/ 2016	44/M	Unknown	frontal brain abscess	Sinusitis	Microaerophilic streptococcus, Strep mitis B	Ceftriaxone x 1 m	Brain biopsy and drainage	Lost to follow-up
Rahiminejad M/2015	50/M	Dysphasia,R-sided facial weakness	L temporal lobe	unable to identify.but periodontal disease	Actinomyces meyeri and Fusobacterium nucleatum.	metronidazole and clindamycin 1 m, Subsequent unclear	twice stereotactic aspiration	abscess recurred after 1 m
Sah R/2020	35/M	HA, L-si ded weakness	R parietal parasa gittal region	dental hygiene	Actinomyces meyeri.	Ampicillin-sulbactam iv 6w and oral 12 m	craniotomy and lesion was excised	No recurrence at 1 year
Shintaku M/2020	72/M	Fever,L-sided hemiparesis	R parietal lobe	Long-term corticosteroid and an immuno- suppressive drug (mizoribine)	a small number of sulfur granules	vancomycin and meropenem iv 3d	none	died of respiratory insufficiency 2 days after admission
Pereira AJDSPR/2022	60/M	HA,barely perceptible speech,L-sided hemiparesis	R posterior parietal cortico-subcortical	dental origin	Actinomyces meyeri and Fusobacterium nucleatum	penicillin and metronidazole iv 3w and orally thereafter	surgical drainage	residual neurological deficits
Our case	39/F	Fever with vomiti Ng,AMS	R tempo ral and parietal lobe	none	none	Vancomycin, meropenem, Ornidazole x3 m	craniotomy, Drainag e	Vegetative state

M: male; F: female; L: Left; R: Right; d: day(s); w: week(s); m: month(s); iv: intravenous;mill U:million units; AMS: altered mental status; HA: headache

Laboratory tests for diagnosing brain abscesses associated with *Actinomyces meyeri* infection lack specificity. Lumbar puncture is not recommended, as it provides limited diagnostic assistance and can potentially induce brain herniation in patients with intracranial hypertension. On the other hand, cranial CT or MRI imaging can aid in the diagnosis, with MRI and diffusion-weighted imaging being more sensitive than CT scans. Actinomycotic brain abscesses manifest as one or more peripherally enhanced lesions on MRI. These lesions may display high-signal edges on T1-weighted non-contrast images and exhibit grape-cluster patterns on T2-weighted low-signal images at the borders [29, 32].

The treatment of *Actinomyces meyeri*-induced brain abscesses requires a combination of antimicrobial drugs, surgical intervention, and elimination of the primary focus of infection. Detecting the etiology of the infection is crucial for the administration of more effective and targeted antimicrobial therapy. Although the positivity rates in CSF and blood sample cultures are relatively low [22], which may be attributed to antibiotic usage and testing methods, the acquisition of specimens for culture and for Gram staining remains particularly important. The replacement of traditional microbiology detection methods by matrix-assisted laser desorption/ionization time-of-flight (MALDI-TOF) technology has the potential to enhance bacteria detection rates. Before utilizing advanced detection technology, it is beneficial to rely on general knowledge about bacteria originating from various sources of infection. This knowledge can help predict the most probable pathogens responsible for the abscess, which in turn can facilitate the selection of the most appropriate antimicrobial therapy. Additionally, it is imperative to select drugs that can readily cross the blood–brain barrier and penetrate the abscess wall. In case of large diameter abscesses, aspiration or surgical removal can be considered. In our case, as the abscess had ruptured into the ventricles and led to brain herniation, craniotomy decompression and removal of the focus of infection were performed. Additionally, drainage tubes were inserted, and mannitol and steroids were administered to reduce intracranial pressure.

The duration of antibiotic treatment for *Actinomyces meyeri* brain abscesses remains unstandardized. A review of the literature indicates that the typical approach to the treatment of *Actinomyces* infections involves administering intravenous penicillin at a daily dose of 18 to 24 million units for a period of 2–6 weeks, followed by oral penicillin or amoxicillin for 6–12 months [22]. For abscesses caused by *Actinomyces meyeri*, the preferred antibiotic regimen consists of penicillin G in combination with metronidazole, with the duration of treatment varying from six weeks to one year. When clinically appropriate, intravenous medication may involve broader spectrum antibiotics such as piperacillin–tazobactam, cefoxitin, ceftriaxone, or carbapenems. In patients that are allergic to penicillin, alternative drugs such as clindamycin, which has good penetration into abscesses, as well as macrolides, doxycycline, tigecycline, and chloramphenicol should be considered [33]. It is important to note that these drugs differ in their ability to penetrate the central nervous system.

Considering the severity of this particular case, which involved subsequent multiple systemic infections, a treatment regimen consisting of meropenem, linezolid, and ornidazole was chosen and administered over three months. This combination provided broad coverage against both aerobic and anaerobic microorganisms. No recurrence of the abscess was registered at the one-year follow-up. Successful treatment of *Actinomyces meyeri*-associated brain abscess has previously been reported with a continuous infusion of 24 million units of benzylpenicillin and 36 million units of dexamethasone as adjunctive for one month. No recurrence was observed in the patient after a year of follow-up [24]. However, the conventional recommendation of 6–12 months of antibiotic treatment may not be suitable for all patients. Moreover, the treatment duration, to some extent, depends on factors such as the initial disease burden, the effectiveness of the surgical removal, and the patient’s response to treatment.

The prognosis of *Actinomyces meyeri*-associated brain abscesses is generally poor, with most patients experiencing residual neurological deficits. Among comatose patients, the mortality rate can be as high as 89% [11]. However, with the advancement of medical science, the mortality rate from cerebral edema during pregnancy has noticeably decreased in recent years, and the overall prognosis has gradually improved. Several factors contribute to the poor prognosis of brain abscesses, including abscess rupture within the ventricles, presence of hydrocephalus, and depth of the abscess. In this particular case, the patient did not receive early diagnosis and treatment, leading to rupture of the abscess into the ventricles. Despite undergoing surgical abscess removal and receiving aggressive anti-infection treatment, the patient experienced significant post-operative hydrocephalus. Although the patient’s life was saved, serious neurological damage ensued. The patient did not regain consciousness after surgery and remained instead in a coma, requiring the induction of labor. The prognosis in cases like these is significantly influenced by the type of bacterial infection, the extent of the brain damage, and the overall response to treatment. Moreover, brain swelling caused by *Actinomyces meyeri* typically exhibits a less pronounced headache, and symptoms such as fever and vomiting can be easily mistaken for early pregnancy reactions, resulting in delayed treatment and poorer prognosis.

## Conclusion

During pregnancy, a decrease in immunity can result in a heightened susceptibility to severe infections, even from bacterial species normally present in the body. Despite the low incidence of *Actinomyces meyeri*-associated brain abscesses during pregnancy, they pose a significant threat to the health of both the mother and fetus, necessitating complex treatment approaches and multidisciplinary collaboration. However, the advancement of neuroimaging techniques, antimicrobial drugs, treatment protocols, and neurosurgical technologies has significantly reduced the mortality rate and led to an overall improved prognosis for patients. In this case, the patient displayed atypical initial symptoms that included only mild fever and vomiting, which were initially misinterpreted as early pregnancy reactions and consequently overlooked, resulting in a missed opportunity for timely intervention. Hence, it is crucial to promptly consult with healthcare professionals when symptoms such as fever, headache, altered mental state, and neurological deficits arise during pregnancy to rule out the possibility of a brain abscess. Prognosis can be further compromised if a brain abscess worsens or if it eventually ruptures into the ventricles, which may lead to brain herniation.

In summary, the rupture of an *Actinomyces meyeri*-associated brain abscess during pregnancy is rare and typically linked to underlying etiologies. Therefore, it is advisable to eliminate potential dental sources of infection and improve oral hygiene before pregnancy. To the best of our knowledge, this is the first reported case of a woman in early pregnancy with an *Actinomyces meyeri*-induced brain abscess in whom no apparent predisposing factors were observed except for dental caries detected on radiographic images. Additionally, the decreased immunity during pregnancy may have heightened the risk of abscess formation and contributed to the atypical clinical course and symptoms of the infection.

### Electronic supplementary material

Below is the link to the electronic supplementary material.

Supplementary Material 1

## Data Availability

The datasets used and/or analyzed during the current study are available from the corresponding author on reasonable request.

## Abbreviations

CT: Computed tomography
CSF: Cerebrospinal fluid
MRI: Magnetic resonance imaging
TIW: T1-weighted
T2W: T2-weighted
DWI: Diffusion weighed imaging

## References

[1] Raskind R (1966). Frontal lobe abscess simulating Stroke in two women, one pregnant. Angiology.

[2] Braham J (1967). EEG in brain abscess. Electroencephalogr Clin Neurophysiol.

[3] Kahn HS, Tyler CW (1975). Mortality associated with use of IUDs. JAMA.

[4] Martinez AJ, Garcia CA, Halks-Miller M, Arce-Vela R (1980). Granulomatous amebic encephalitis presenting as a cerebral mass lesion. Acta Neuropathol (Berl).

[5] Wolf P, Simon M (1983). Dimethyl sulphoxide (DMSO) induced serum hyperosmolality. Clin Biochem.

[6] Opsahl MS, O’Brien WF (1983). Systemic nocardiosis in pregnancy. A case report. J Reprod Med.

[7] Braun TI, Kerson LA, Eisenberg FP (1991). Nocardial brain abscesses in a pregnant woman. Rev Infect Dis.

[8] Lee CN, Wu CC, Lin PY, Hsieh FJ, Chen HY (1994). Pregnancy following cardiac prosthetic valve replacements. Obstet Gynecol.

[9] Cihangiroglu M, Hartker FW, Mojtahadi S, Ramsey RG (2001). Intracranial vasculitis and multiple abscesses in a pregnant woman. J Neuroimaging.

[10] Baxi LV, Mayer SA, Mansukhani M (2001). Cerebral abscess and thrombophilia in pregnancy. A case report. J Reprod Med.

[11] Wax JR, Blackstone J, Mancall A, Cartin A, Pinette MG (2004). Sinogenic brain abscess complicating pregnancy. Am J Obstet Gynecol.

[12] Liu SSH, Chen SH, Chang KC, Wang JP (2004). Brain abscess presenting as postpartum Diabetes insipidus. Taiwan J Obstet Gynecol.

[13] Kim HC, Kang SG, Huh PW, Yoo do S, Cho KS, Kim DS (2007). Pituitary abscess in a pregnant woman. J Clin Neurosci.

[14] Jacob CE, Kurien M, Varghese AM, Aleyamma TK, Jasper P, Prabu K (2009). Treatment of otogenic brain abscess in pregnancy. Otol Neurotol.

[15] Salvi N, Massiah N, Nuttall ID (2010). Cerebral abscess in pregnancy: a clinical dilemma. Arch Dis Child Fetal Neonatal Ed.

[16] Hobson DT, Imudia AN, Soto E, Awonuga AO (2011). Pregnancy complicated by recurrent brain abscess after extraction of an infected tooth. Obstet Gynecol.

[17] Yoshida M, Matsuda H, Furuya K (2013). Successful prognosis of brain abscess during pregnancy. J Reprod Infertil.

[18] Bulthuis VJ, Gubler FS, Teernstra OP, Temel Y (2015). A case of a brain stem abscess with a favorable outcome. Surg Neurol Int.

[19] Jendoubi A, Aissa S, Dridi R, Ammari L, Houissa M (2016). Brain abscess during pregnancy mimicking eclampsia: a diagnostic and therapeutic challenge. Anaesth Crit Care Pain Med.

[20] Bahrami R, Safari H (2017). A fatal sinogenic brain abscess in pregnancy: Case Report and Review of Literature. Indian J Neurosurg.

[21] Kononen E, Wade WG (2015). Actinomyces and related organisms in human Infections. Clin Microbiol Rev.

[22] Sah R, Nepal G, Sah S, Singla S, Upadhyay P, Rabaan AA (2020). A rare case of brain abscess caused by Actinomyces meyeri. BMC Infect Dis.

[23] Clancy U, Ronayne A, Prentice MB, Jackson A (2015). Actinomyces meyeri brain abscess following dental extraction. BMJ Case Rep.

[24] Dijkmans BA, Thomeer RT, Vielvoye GJ, Lampe AS, Mattie H (1984). Brain abscess due to Streptobacillus moniliformis and actinobacterium meyeri. Infection.

[25] Kuijper EJ, Wiggerts HO, Jonker GJ, Schaal KP, de Gans J (1992). Disseminated actinomycosis due to Actinomyces meyeri and Actinobacillus actinomycetemcomitans. Scand J Infect Dis.

[26] Park HJ, Park KH, Kim SH, Sung H, Choi SH, Kim YS (2014). A case of disseminated Infection due to actinomyces meyeri involving lung and brain. Infect Chemother.

[27] Fernandez-Valle T, Guío Carrión L, Galbarriatu Gutiérrez L (2014). Vilar Achabal B. [Actinomyces meyeri brain abscess]. Med Clin (Barc).

[28] Rolfe R, Steed LL, Salgado C, Kilby JM (2016). Actinomyces meyeri: a common agent of actinomycosis. Am J Med Sci.

[29] Rahiminejad M, Hasegawa H, Papadopoulos M, MacKinnon A (2016). Actinomycotic brain abscess. BJR Case Rep.

[30] Shintaku M, Kono F, Ando K, Kobayashi Y, Hasegawa H, Tsutsumi Y (2020). Acute actinomycotic brain abscess in a patient with rheumatoid arthritis. Neuropathology.

[31] Pereira AJDSPR, Tavares AT, Prates M, Ribeiro N, Fonseca LF, Marques MDR (2022). Brain abscess: a rare clinical case with oral etiology. Case Rep Infect Dis.

[32] Heo SH, Shin SS, Kim JW, Lim HS, Seon HJ, Jung SI (2014). Imaging of actinomycosis in various organs: a comprehensive review. Radiographics.

[33] Gajdács M, Urbán E, Terhes G (2019). Microbiological and clinical aspects of Cervicofacial Actinomyces Infections: an overview. Dent J.

