# Supplementary material for: Actinomyces meyeri-induced brain abscess in pregnancy: a case report
Source: BMC Neurol. 2023 Nov 11;23:401. doi: 10.1186/s12883-023-03453-7 (PMC10638749; doi:10.1186/s12883-023-03453-7)
Supplement: Supplementary file 1 — Supplementary Material 1 [file 12883_2023_3453_MOESM1_ESM.pdf]

CARE
case report guidelines

CARE Checklist of information to include when writing a case report

(cc) BY-NC-ND

Topic	Item	Checklist item description	Reported on Line
Title	1	The diagnosis or intervention of primary focus followed by the words "case report"	Page1/line1
Key Words	2	2 to 5 key words that identify diagnoses or interventions in this case report, including "case report"	Page3/line52
Abstract (no references)	3a	Introduction: What is unique about this case and what does it add to the scientific literature?	Page2-3/line43-50
	3b	Main symptoms and/or important clinical findings	Page4/line69-79
	3c	The main diagnoses, therapeutic interventions, and outcomes	Page5-6/line98-120
	3d	Conclusion—What is the main "take-away" lesson(s) from this case?	Page12/line251-258
Introduction	4	One or two paragraphs summarizing why this case is unique (**may include references**)	Page12-13/line245-266
Patient Information	5a	De-identified patient specific information.	Page3/line69
	5b	Primary concerns and symptoms of the patient.	Page4/line69-74
	5c	Medical, family, and psycho-social history including relevant genetic information	Page7/line149-153
	5d	Relevant past interventions with outcomes	Page7/line140-141
Clinical Findings	6	Describe significant physical examination (PE) and important clinical findings.	Page4-5/line74-105
Timeline	7	Historical and current information from this episode of care organized as a timeline	NA
Diagnostic Assessment	8a	Diagnostic testing (such as PE, laboratory testing, imaging, surveys).	Page5/line99-105
	8b	Diagnostic challenges (such as access to testing, financial, or cultural)	Page9/line187-195
	8c	Diagnosis (including other diagnoses considered)	Page4/line84-86
	8d	Prognosis (such as staging in oncology) where applicable	Page10-11/line226-242
Therapeutic Intervention	9a	Types of therapeutic intervention (such as pharmacologic, surgical, preventive, self-care)	Page9/line184-190
	9b	Administration of therapeutic intervention (such as dosage, strength, duration)	Page10-11/line202-225
	9c	Changes in therapeutic intervention (with rationale)	NA
Follow-up and Outcomes	10a	Clinician and patient-assessed outcomes (if available)	Page6/line116-120
	10b	Important follow-up diagnostic and other test results	Page6/line117-120
	10c	Intervention adherence and tolerability (How was this assessed?)	NA
	10d	Adverse and unanticipated events	NA
Discussion	11a	A scientific discussion of the strengths AND limitations associated with this case report	Page10/line 198-201
	11b	Discussion of the relevant medical literature **with references**.	Page6-11/line123-228
	11c	The scientific rationale for any conclusions (including assessment of possible causes)	Page12-13/line258-266
	11d	The primary "take-away" lessons of this case report (without references) in a one paragraph conclusion	Page12/line251-258
Patient Perspective	12	The patient should share their perspective in one to two paragraphs on the treatment(s) they received	NA
Informed Consent	13	Did the patient give informed consent? Please provide if requested	Yes ☐ No ☑
